# *Scorpiops ingens* sp. n. and an updated key to the *Scorpiops* from China (Scorpiones, Euscorpiidae, Scorpiopinae)

**DOI:** 10.3897/zookeys.495.9085

**Published:** 2015-04-08

**Authors:** Shijin Yin, Yunfeng Zhang, Zhaohui Pan, Shaobin Li, Zhiyong Di

**Affiliations:** 1 College of pharmacy, South Central University for Nationalities, Wuhan 430074, P.R. China; 2 Department of Life Science, Tangshan Normal University, Tangshan 063000, P.R. China; 3 Institute of Plateau Ecology, Agriculture and Animal Husbandry College of Tibet University, Linzhi 860000, P.R. China; 4 College of Life Sciences, Yangtze University, Jingzhou 434025, P.R. China; 5 School of Life Sciences, University of Science and Technology of China, Hefei 230027, P.R. China

**Keywords:** China, Euscorpiidae, scorpion, *Scorpiops*, Xizang

## Abstract

A new species, *Scorpiops ingens* **sp. n.**, from Xizang, is described and illustrated. *Scorpiops ingens* **sp. n.** is characterized by yellow-brown color, large size (length of adults above 70.0 mm), small and dense granules on tegument, a pair of small median eyes, 17 external trichobothria (5 *eb*, 2 *esb*, 2 *em*, 4 *est*, 4 *et*), and 7 or 8 (usually 7) ventral trichobothria in the pedipalp patella, chela with a length/width ratio average of 2.2 in males and females, pedipalp chela fingers on adult females and males scalloped, pectinal teeth count 6–8, pectinal fulcra absent. With the description of this new species, the number of known species of *Scorpiops* from China is raised to 12. An updated identification key to *Scorpiops* from China is presented.

## Introduction

Recently, the diversity of *Scorpiops* species from China was highlighted (Qi et al. 2005; Di and Zhu 2009; Di et al. 2011; 2014). Kovařík and Ahmed (2009) referred to an unresolved, widespread *Scorpiops hardwickii* “complex” (12 species) which, in their opinion, included five species known from China (*Scorpiops atomatus*, *Scorpiops hardwickii*, *Scorpiops langxian*, *Scorpiops pococki*, and *Scorpiops tibetanus*). Di et al. (2011) suggested that *Scorpiops atomatus* and *Scorpiops tibetanus* should be excluded from *Scorpiops hardwickii* “complex” by morphological analysis results, and provided unifying features of *Scorpiops hardwickii* “complex” base on the species from China: (1) color red-brown to dark brown; (2) total length approximately 45–80 mm in adults; (3) fingers of pedipalps very strongly flexed (curved) in males, slightly flexed (undulated) in females; (4) ventral trichobothria on patella number 6–8; (5) pectinal teeth number 4–9; (6) length/width ratio of chela about 1.8–2.1; (7) fulcra absent; (8) patella with two small spinoid granules on the internal aspect. There are three species from China belonging to *Scorpiops hardwickii* “complex” after the revision provided by Di et al. (2011): *Scorpiops hardwickii*, *Scorpiops langxian*, and *Scorpiops pococki*. In fact, Qi et al. (2005: 29) pointed out the differences among *Scorpiops hardwickii*, *Scorpiops langxian*, and *Scorpiops pococki*: pedipalp chela manus almost as long as wide in *Scorpiops hardwickii*, while the pedipalp chela manus usually longer than its width in *Scorpiops langxian* and *Scorpiops pococki*; distance between median eyes much more than their diameter in *Scorpiops langxian*, while the distance between median eyes only slightly more than their diameter in *Scorpiops pococki*. Di et al. (2014) recorded 11 species in the updated checklist of scorpions from China based mainly on the literature: *Scorpiops atomatus* (Xizang), *Scorpiops hardwickii* (Xizang), *Scorpiops jendeki* (Yunnan), *Scorpiops langxian* (Xizang), *Scorpiops leptochirus* (Xizang), *Scorpiops lhasa* (Xizang), *Scorpiops luridus* (Xizang), *Scorpiops margerisonae* (Xizang), *Scorpiops petersii* (Xizang), *Scorpiops pococki* (Xizang), and *Scorpiops tibetanus* (Xizang).

## Material and methods

Identification and measurements were made using a Motic K700 stereomicroscope with an ocular micrometer. The photos were taken with a Canon (650D) camera. Measurements follow Sissom et al. (1990) and are given in mm. Trichobothrial notations follow Vachon (1974) and morphological terminology mostly follows Hjelle (1990). Research materials have been deposited in the Specimen Room of University of Science and Technology of China, Hefei, China (USTC).

## Taxonomy

### Family Euscorpiidae Laurie, 1896 Subfamily Scorpiopinae Kraepelin, 1905 Genus Scorpiops Peters, 1861

#### Scorpiops ingens sp. n.

Taxon classificationAnimaliaScorpionesEuscorpiidae

http://zoobank.org/D662B45D-6871-419F-9D3A-31EE4C53559D

Figs 1–4 , 5–8 , 9–18

##### Type material.

Holotype male (USTC), China: Xizang, Lhasa banlieue, 26/VII/2014, Zhiyong Di leg. (Ar.-USTC-XZLS1401); paratypes: 1 adult female, 1 immature female, and 1 juvenile male, same data as holotype (Ar.-USTC-XZLS1402–1404) (kept in USTC).

##### Diagnosis.

In accordance with the grouping of species proposed by Kovařík (2000) for the genus *Scorpiops*, the new species, which has 7 (rarely 8) trichobothria on the ventral surface of the patella, has to be placed in the *Scorpiops hardwickii* “complex” group. The new species differs from other members of the group in having yellow-brown color, larger size (length of adults above 70.0 mm), small and dense granules on the tegument, a pair of small median eyes and a lofty median ocular tubercle.

*Comments*. There are four close relatives from China distributed near to *Scorpiops ingens* sp. n.: *Scorpiops hardwickii*, *Scorpiops langxian*, *Scorpiops petersii*, and *Scorpiops pococki*. But *Scorpiops hardwickii*, *Scorpiops langxian*, and *Scorpiops pococki* with red-brown to black-brown color, body length no longer than 65 mm. Although *Scorpiops petersii* also above 75.0 mm, its carapace is not densely granulated, granules on its mesosoma are widely spaced, with the distance between them far greater than their size (Kovařík 2000: 193), while the granules are dense on the carapace and mesosoma of *Scorpiops ingens* sp. n.

##### Etymology.

The specific name refers to the size of the morphology of the new species.

##### Description.

Based on male holotype and female paratype.

**Figures 1–4.**
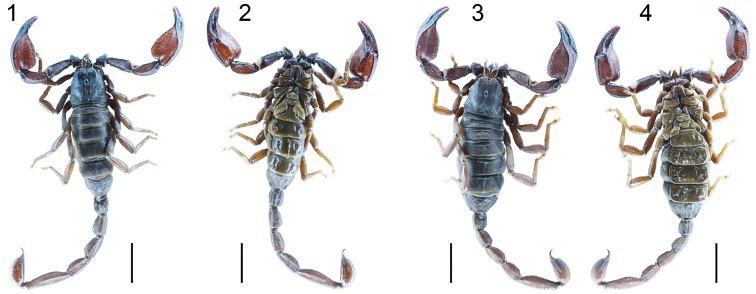
Habitus of *Scorpiops ingens* sp. n. Dorsal and ventral habitus: **1–2** Male holotype (Ar.-USTC-XZLS1401) **3–4** Female paratype (Ar.-USTC-XZLS1402). Scale bar = 10.0 mm.

**Figures 5–8.**
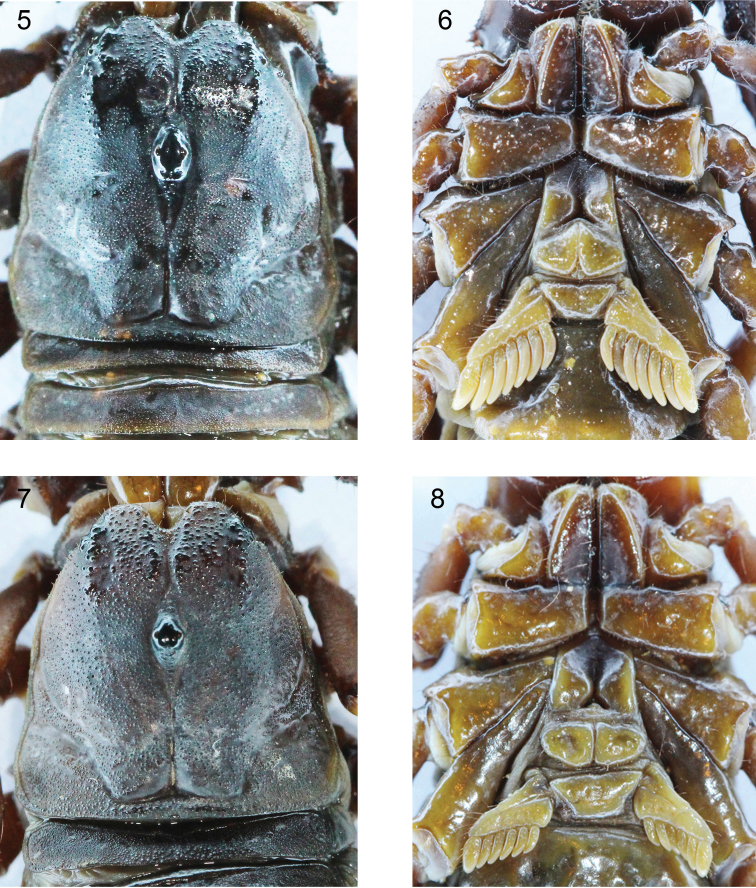
*Scorpiops ingens* sp. n. Male holotype: **5, 6. 5** Carapace and tergite I− II. **6** Ventral aspect of prosoma. Female paratype (Ar.-USTC-XZLS1402): **7, 8. 7** Carapace and tergite I **8** Ventral aspect of prosoma.

*Coloration*. Mostly yellow to yellow-brown (Figs 1–4). Carapace yellow-brown with unconspicuous dark stripe (Figs 5, 7), median and lateral ocular tubercles black. Tergites and metasoma segments yellow-brown. Vesicle yellow-brown with a dark brown aculeus. Chelicerae yellow-brown, with fingers black brown and gradually lighter toward the tip. Pedipalp yellow-brown, with the carinae black-brown. Legs yellow-brown. Claws yellow-brown with brown tips. Sternum and sternites yellow-brown (Figs 6, 8). Genital operculum, basil piece and pectines yellow (Figs 6, 8).

*Morphology*. Prosoma: Carapace coarse, with sparse and large granules in the area of the front of the eye region, with dense and minute granules in the area of the behind of the eye region; lateral furrow broad and flat; anterior median furrow broad and deep; posterior median furrow deep; anterior margin nearly smooth; posterior and lateral margins and other parts with dense, minute granules (Figs 5, 7). Median eyes small and same as the first lateral eye, situated anterior to the center of the carapace; three pairs of lateral eyes, the third smallest. Median ocular tubercle high and smooth, with a median furrow, which having some granules. Lateral ocular tubercle with some big smooth granules.

*Mesosoma*: Tergites are almost completely densely covered with equal minute granules in male holotype, posterior part with some bigger granules in female paratypes; from tergite II to VI the trace of a median carina first appears and gradually becomes distinct; on tergite VII with a distinct apophysis and two pairs of lateral carinae. Sternum pentagonal (Figs 6, 8). Pectinal teeth count 6–8 (rarely 8), fulcra absent (Figs 6, 8). Genital opercula subtriangular (Figs 6, 8). Sternites smooth and shiny (Figs 2, 4); segment VII ventrally with four weak carinae.

*Metasoma*: Tegument coarse. Segments I to V are longer than wide; segments I to V have 10-8-8-8-7 carinae, segments II–IV with a pair of vestigial lateral carinae; all carinae granular; on segment V, ventral carinae with larger serration. Vesicle smooth, with some granules and few setae.

*Chelicerae*: Tibiae smooth. Movable finger with 4 denticles on dorsal edge and 6 denticles on ventral edge (smaller in female). Fixed finger with 3 denticles on dorsal edge.

**Figures 9–18.**
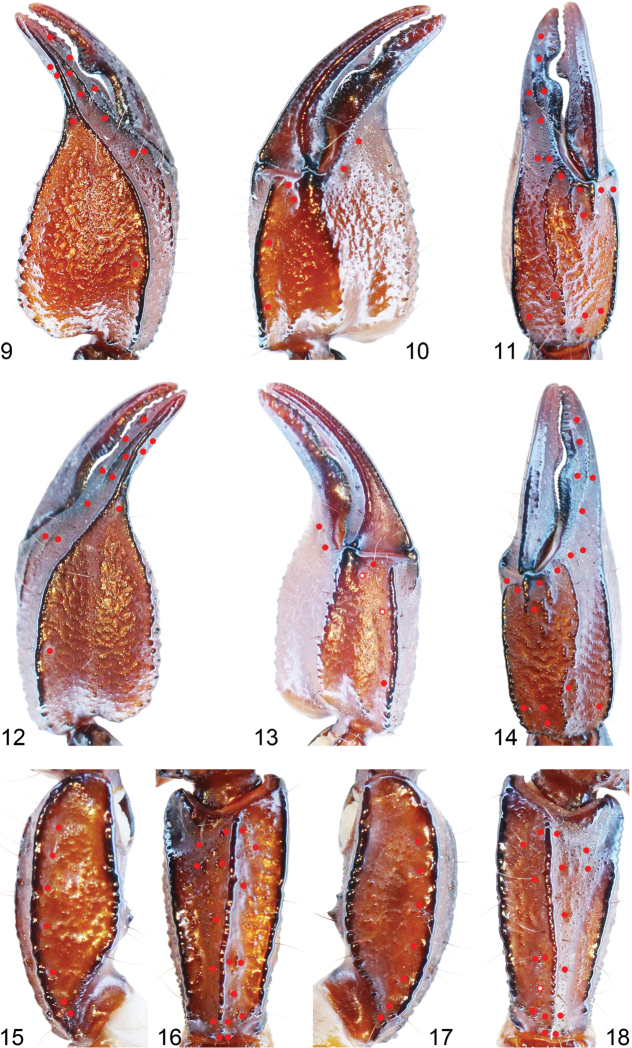
*Scorpiops ingens* sp. n. Male holotype: **9–11, 15–16. 9**–**11** Chela (right) dorsal and external, ventral and internal, and external aspects. **15–16** Patella (right) ventral and external aspects. Female paratype (Ar.-USTC-XZLS1402): **12–14, 17–18. 12–14** Chela (left) dorsal and external, ventral and internal, and external aspects. **17–18** Patella (left) ventral and external aspects. The red dots and rings denote trichobothrial patterns of pedipalps, the red ring meaning vestigial.

*Pedipalps*: Tegument of femur and patella coarse, tegument of chelae and ventral aspects of femur and patella smooth. Femur with dorsointernal, dorsoexternal, external, ventroexternal, ventrointernal carinae granulated, and internal carinae crenulated. Patella with dorsoexternal, dorsointernal, external, ventrointernal, ventroexternal carinae with large, smooth granules; two small spinoid granules present on the internal aspect. Trichobothrial pattern C, neobothriotaxic; patella with 17 external trichobothria (5 eb, 2 esb, 2 em, 4 est, 4 et) and 7 or 8 (usually 7) ventral trichobothria (Figs 15–18). Chela with 4 ventral trichobothria, with dorsal marginal, external secondary, and ventral internal carinae, all smooth; internal carina vestigial only with few large granules (Figs 9–14). Male pedipalp chela fingers stronger curved than females.

*Legs*: Tegument coarse except coxa and trochanter. Trochanter with few granules and setae. Femur dorsal surface densely granular and ventrally smooth, internally with 2 granular carinae. Patella dorsal surface densely granular and ventrally smooth, with dorsoexternal, dorsal and ventroexternal granular carinae. Tibiae with few setae, without spurs. Basitarsus with more setae, and two lateral pedal spurs. Tarsus ventrally with row of spinules. Ungues falcate.

##### Variation.

Female and male: coloration and morphology are very similar. Number (left/right) of ventral trichobothria on the pedipalp patellae: two females with 8/7 and 7/7, two males with 7/7. Number of pectinal teeth: two females with 6/6, two males with 7/7 and 7/8. Measurements in Table 1.

**Table 1.** Measurements (in mm) of holotype (male, Ar.-USTC-XZLS1401) and paratype (female, Ar.-USTC-XZLS1402) of *Scorpiops ingens* sp. n.

	*Scorpiops ingens* sp. n.	
	Holotype	Paratype
Total length:	74.6	75.9
Carapace: -Length -Anterior width -Posterior width	8.7 4.9 8.9	9.6 5.3 9.8
Mesosomal segments: -Length	22.3	24.7
Metasomal segment I: -Length -Width -Depth	4.6 4.0 3.1	4.5 4.1 3.2
Metasomal segment II: -Length -Width -Depth	5.1 3.6 3.0	5.2 3.7 2.9
Metasomal segment III: -Length -Width -Depth	6.3 3.3 3.0	5.5 3.5 3.0
Metasomal segment IV: -Length -Width -Depth	6.8 3.0 3.0	6.2 3.3 3.0
Metasomal segment V: -Length -Width -Depth	10.8 3.0 3.0	10.6 3.0 3.2
Telson: -Length -Width -Depth	10.0 3.8 3.7	9.6 3.6 3.6
Pedipalp femur: -Length -Width -Depth	7.0 3.1 2.6	8.1 3.3 2.9
Pedipalp patella: -Length -Width -Depth	7.0 3.1 3.3	7.6 3.5 3.6
Chela: -Length -Width (manus) -Depth (manus)	14.3 6.3 4.8	14.8 6.4 5.0
Movable finger: -Length	9.0	9.4
Pectinal teeth (left/right)	7/7	6/6

##### Habitat.

Under stones on a hillside with ruderal vegetation.

##### Distribution.

China (Xizang).

### Updated key to species of *Scorpiops* from China

**Table:** 

1	Fingers of pedipalps are straight or only slightly curved in both sexes	**2**
–	Fingers of pedipalps are curved in both sexes	**3**
2	Ventral trichobothria on patella number 6 (7 rarely), total length 30.0–42.1 mm, pectinal teeth number 4–5, chela length to width ratio about 2.2	***Scorpiops jendeki***
–	Ventral trichobothria on patella number 7, total length 40.0–58.0 mm, pectinal teeth number 7–9, chela length to width ratio about 3.3–3.5	***Scorpiops leptochirus***
3	Manus length to width ratio visibly higher than 1	**4**
–	Manus with similar length and width	**9**
4	Total length more than 65.0 mm	**5**
–	Total length less than 65.0 mm	**6**
5	Ventral patella of pedipalps with 9 trichobothria	***Scorpiops luridus***
–	Ventral patella of pedipalps with 7 (rarely 6 or 8) trichobothria	***Scorpiops petersii***
6	Dorsally flat manus of pedipalps and chela of both sexes, with length/width ratio: 2.1–2.2 (about 2.1 in males and 2.2 in females), total length 40.0–50.0 mm in adults	***Scorpiops margerisonae***
–	Dorsally round manus of pedipalps or at least the chela of one sex, with length to width ratio higher than 2.2 or total length higher than 50.0 mm	**7**
7	Total length less than 40.0 mm	**8**
–	Total length 45.0–61.0 mm	***Scorpiops tibetanus***
8	Chela of pedipalp length to width ratio about 2.6–3.0, dorsal surface of chela of pedipalp coarse	***Scorpiops lhasa***
–	Chela of pedipalp length to width ratio lower than 2.5, dorsal surface of chela of pedipalp smooth with luster	***Scorpiops atomatus***
9	Yellow-brown color, length of adults above 70.0 mm	***Scorpiops ingens* sp. n.**
–	Red-brown color, length of adults under 65.0 mm	**10**
10	Pedipalp chela manus almost as long as wide	***Scorpiops hardwickii***
–	Pedipalp chela manus usually longer than its width	**11**
11	Distance between median eyes much more than their diameter	***Scorpiops langxian***
–	Distance between median eyes only slightly more than their diameter	***Scorpiops pococki***

## Supplementary Material

XML Treatment for Scorpiops ingens

## References

[B1] Di ZY He YW Cao ZJ Wu YL Li WX (2011) The first record of the family Euscorpiidae (Arachnida: Scorpiones) from Central China, with a key of Chinese species of the genus *Scorpiops*. Euscorpius 116: 1–11.

[B2] Di ZY Yang ZZ Yin SJ Cao ZJ Li WX (2014) History of study, updated checklist, distribution and key of scorpions (Arachnida: Scorpiones) from China. Zoological Research 35: 3–19. 24470450 10.11813/j.issn.0254-5853.2014.1.003 PMC5042949

[B3] Di ZY Zhu MS (2009) One new species of the Genus *Scorpiops* Peters, 1861 (Scorpiones: Euscorpiidae, Scorpiopinae) from Xizang, China. Zootaxa 2030: 39–48.

[B4] Hjelle JT (1990) Anatomy and morphology. In: Polis GA (Ed.) The Biology of Scorpions. Stanford Univ. Press, 9–63.

[B5] Kovařík F (2000) Revision of family Scorpiopidae (Scorpiones), with descriptions of six new species. Acta Societatis Zoologicae Bohemicae 64: 153–201.

[B6] Kovařík F Ahmed Z (2009) Three new species of *Scorpiops* Peters, 1861 (Scorpiones: Euscorpiidae: Scorpiopinae) from Pakistan. Euscorpius 88: 1–11.

[B7] Qi JX Zhu MS Lourenco WR (2005) Eight new species of the genera *Scorpiops* Peters, *Euscorpiops* Vachon, and *Chaerilus* Simon (Scorpiones: Euscorpiidae, Chaerilidae) from Tibet and Yunnan, China. Euscorpius 32: 1–40.

[B8] Sissom WD Polis GA Watt DD (1990) Field and laboratory methods. In: Polis GA (Ed.) The Biology of Scorpions. Stanford University Press, Stanford, California, 445–461.

[B9] Vachon M (1974) Etude des caracteres utilises pour classer les familles et les genres de Scorpions (Arachnides). 1. La trichobothriotaxie en arachnologie. Sigles trichobothriaux et types de trichobothriotaxie chez les Scorpions. Bulletin du Museum national d’Histoire naturelle, Paris, 3e ser., 140: 857–958.

